# Developing vaccinology expertise for Africa: fifteen years and counting

**DOI:** 10.11604/pamj.2021.38.313.26744

**Published:** 2021-03-29

**Authors:** Edina Amponsah-Dacosta, Rudzani Muloiwa, Charles Shey Wiysonge, Michael Gold, Gregory Hussey, Benjamin Mugo Kagina

**Affiliations:** 1 Vaccines for Africa Initiative, School of Public Health and Family Medicine, Faculty of Health Sciences, University of Cape Town, Cape Town, South Africa,; 2 Department of Paediatrics and Child health, Faculty of Health Sciences, University of Cape Town, Cape Town, South Africa,; 3 Cochrane South Africa, South African Medical Research Council, Cape Town, South Africa,; 4 Discipline of Paediatrics, School of Medicine, University of Adelaide, Adelaide, Australia,; 5 Institute of Infectious Diseases and Molecular Medicine, Faculty of Health Sciences, University of Cape Town, Cape Town, South Africa

**Keywords:** Child health, health education and promotion, immunization, public health, vaccines

## Abstract

For 15 years, the Annual African Vaccinology Course (AAVC) hosted by the Vaccines for Africa Initiative, has been at the forefront of vaccinology training in Africa. The AAVC was developed in 2005 in response to the growing demand for vaccinology training in Africa. To date, 958 policy makers, immunization managers, public and private health practitioners, scientists, postgraduate and postdoctoral students have been trained. These participants are from 44 of the 54 African countries. The course content covers diverse topics such as considerations for new vaccine introduction, mathematical modelling, and emerging and re-emerging vaccine preventable diseases. As the landscape of vaccinology continues to evolve, the AAVC aims to expand the reach of vaccinology training using blended learning approaches which will incorporate online and face-to-face formats, while expanding access to this popular course. Ultimately, the AAVC endeavours to develop a big pool of vaccinology expertise in Africa and to strengthen regional ownership for immunization programmes.

## Introduction

Immunization programmes in Africa have achieved remarkable progress since the introduction of the Expanded Programme on Immunization (EPI) in 1974 [1]. However, the progress is not as remarkable when compared to other continents with respect to introduction of new vaccines, elimination of vaccine preventable diseases (VPDs), and reduction of morbidity and mortality due to infectious diseases. Variable vaccination coverage due to inequitable distribution of resources means that a significant proportion of infants and children are still at risk of potentially fatal VPDs [2,3]. The substantial cost of new vaccines has also impacted on availability especially in self-financing countries. Reasons for the sub-optimal performance of immunization programmes in Africa are multi-pronged and can be linked with broader health systems factors, including the capacity of the health workforce, the quality of service delivered and the negative impact of public health emergencies such as the COVID-19 pandemic [4]. The level of knowledge and quality of practices of immunization providers are key determinants of vaccine acceptance and effectiveness of an immunization programme. Education and training in vaccinology has been shown to improve immunization practices [5]. It is for this purpose that the Vaccines for Africa Initiative (VACFA), based at the University of Cape Town (UCT) in South Africa, established the Annual African Vaccinology Course (AAVC) in 2005. In addition to delivering basic vaccinology training, the AAVC fosters continuous knowledge exchange among various cadres working in different capacities within national immunization programmes across Africa [6].

## Methods

**Aims of the course:** the aim of the AAVC is to: (i) broaden understanding of the challenges and opportunities in vaccinology at regional and global levels, (ii) equip participants with essential expertise to support national immunization programmes, (ii) build sustainable research capacity for vaccine development including the conduct of high quality phase I-IV vaccine trials in Africa, (iv) explore the roles and challenges of National Immunization Technical Advisory Groups (NITAGs) in strengthening immunization policies in Africa, and (v) foster collaboration and networking among African vaccinologists.

**Course content:** since the inception of the AAVC in 2005, the course content has evolved in response to the rapidly growing field of vaccinology and the increasing demand for developing vaccinology expertise in Africa. Further information on the AAVC course content may be sourced from the VACFA website [7]. Briefly, the course is delivered over a period of five days and covers the basic sciences, such as epidemiology, immunology and microbiology, pertaining to VPDs. In addition to this, the course content includes key issues on vaccine development, including new vaccines in the pipeline, vaccine safety, vaccination strategies, evaluation of vaccines and vaccination programmes, composition of NITAGs as well as the functions of these advisory bodies, and promotion of vaccine advocacy and communication. The course content has expanded in recent years to include key concepts on mathematical modelling for control of VPDs, emerging and re-emerging infectious diseases such as Ebola and Zika, health systems strengthening to support immunization programmes, and the application of evidence-based practices in vaccinology.

**Format of the course:** this is usually a five-day face-to-face course taking place in the last quarter of the year. While previous versions of the AAVC were held in several African countries [6], this was changed in 2009 to a residential format, which has since been held each year in Cape Town, South Africa. The change in format was necessary in order to maximize the reach of funding earmarked for the course by accommodating a larger participant cohort and inviting keynote speakers or faculty members to the course. Recognizing the importance of blended learning approaches in enhancing training outcomes, the AAVC organizing committee in 2017, introduced an online vaccinology pre-course and evaluation for participants. The online pre-course is a collaborative initiative between VACFA and a non-profit immunology educational organization, Immunopaedia [8]. The online pre-course is designed to assess participants´ knowledge of core vaccinology concepts and prime them on the course content prior to attending the face-to-face format of the AAVC.

As part of VACFA´s initiative to strengthen the vaccine policy and decision-making landscape in Africa, the scope of the AAVC was extended in 2018 to include a workshop dedicated to exploring the roles of, and challenges faced by NITAGs. The workshop is a networking opportunity and explores possible technical and scientific support mechanisms for NITAGs in Africa. In its first offering during the 14^th^ AAVC in 2018, the NITAG workshop was held over 1.5 days. Attendees of the workshop included core members and secretariat of NITAGs from nine African countries, namely; Angola, Cameroon, Ghana, Nigeria, Rwanda, South Africa, Tanzania, Togo and Uganda. Discussions were facilitated by representatives from VACFA and the World Health Organization (WHO) [9]. This was a unique platform for knowledge exchange as NITAG members shared country experiences, including achievements and challenges faced in developing evidence-based recommendations. The recommendations are aimed at strengthening national immunization programmes. Impressively, discussions of this workshop included the development of new strategies to continuously support NITAGs within the African continent.

## Results

**Figure 1:**
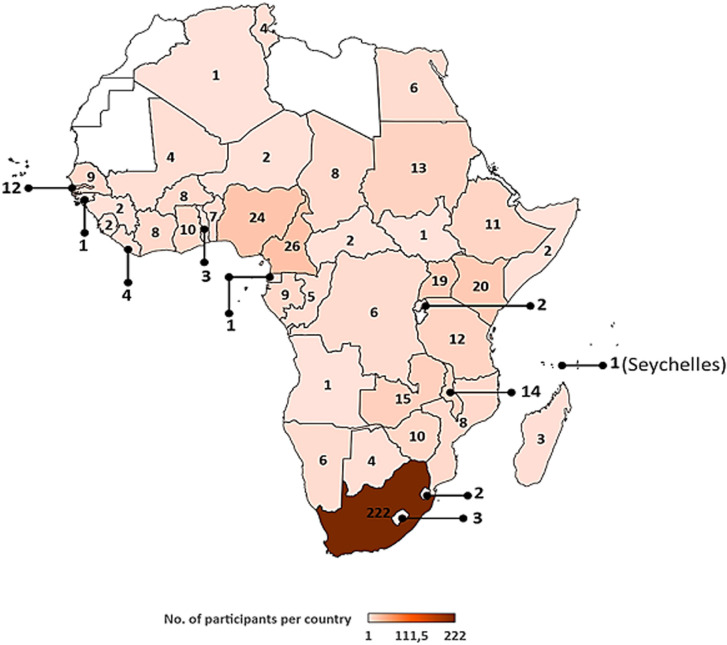
origin of participants of the Annual African Vaccinology Course between 2011 and 2019

**Course participants:** a report on the participants and faculty members of the AAVC held between 2005 and 2010 was previously published [6]. Between 2011 and 2019, about 1560 applications were received from various African countries. Table 1 provides a breakdown of the number of applications received each year and an indication of the application success rate. On average, 173 applications are received each year and about 60 applicants are selected to participate in the course. The screening process for the applications involves an independent review by the AAVC organizing committee. Applications are assigned scores based on applicants´ decision-making responsibilities or involvement in national or sub-national immunization programmes, motivation to attend the course, and a recommendation by line-managers. The AAVC organizing committee then meets to resolve any disparities in the scoring process through discussion and consensus. Thereafter, successful applicants are informed of the outcome of their application. Since the inception of the AAVC, the conveners have endeavoured to extend the reach of the course to as many participants as possible in order to bridge the gap in training needs within the continent. For this reason, participants are only allowed to attend the AAVC once. In addition, at least one participant is selected from each country bearing all other selection criteria in mind. Given that participation at the AAVC is fully funded, the number of participants attending each year is also dependent on the availability of funding to cover flights, accommodation, venue hire, meals and course materials. Since 2011, 538 individuals have participated in the AAVC, bringing the total number of participants trained between 2005 and 2019, to 958. The total number excludes walk-in participants who are typically UCT-affiliated and based in Cape Town. Participants of the AAVC have included members of NITAGs, national and sub-national EPI managers, public and private health practitioners (including nurses and medical doctors), scientists (researchers in immunology, vaccinology, or related fields), postgraduate and postdoctoral students, individuals working with non-governmental agencies like Médecins Sans Frontières (MSF), and pharmaceutical companies (including GlaxoSmithKline Biologicals [GSK], Merck Sharp & Dohme [MSD], Pfizer, and Sanofi Pasteur). To date, the AAVC has trained participants from 44 of the 54 African countries (Figure 1). Only Burundi, Cape Verde, Comoros, Djibouti, Eritrea, Libya, Mauritania, Morocco, Sao Tome and Principe, and the Western Sahara are yet to participate in the course. To some extent, the lack of participation from these countries may reflect language barriers given that the AAVC is conducted in English. This underscores the need to develop vaccinology courses for non-Anglophone countries or to design courses that are inclusive of the diverse official languages represented within Africa.

**Table 1:** summary of the AAVC application success rate between 2011 and 2019

Year	No. of applications	No. of successful applicants (%)
2011	136	59 (43.4%)
2012	228	64 (28.1%)
2013	107	49 (45.8%)
2014	141	66 (46.8%)
2015	172	57 (33.1%)
2016	209	49 (23.4%)
2017	120	74 (61.7%)
2018	280	61 (21.8%)
2019	167	59 (35.3%)
**Total**	1560	538 (34.5%)

**Figure 2:**
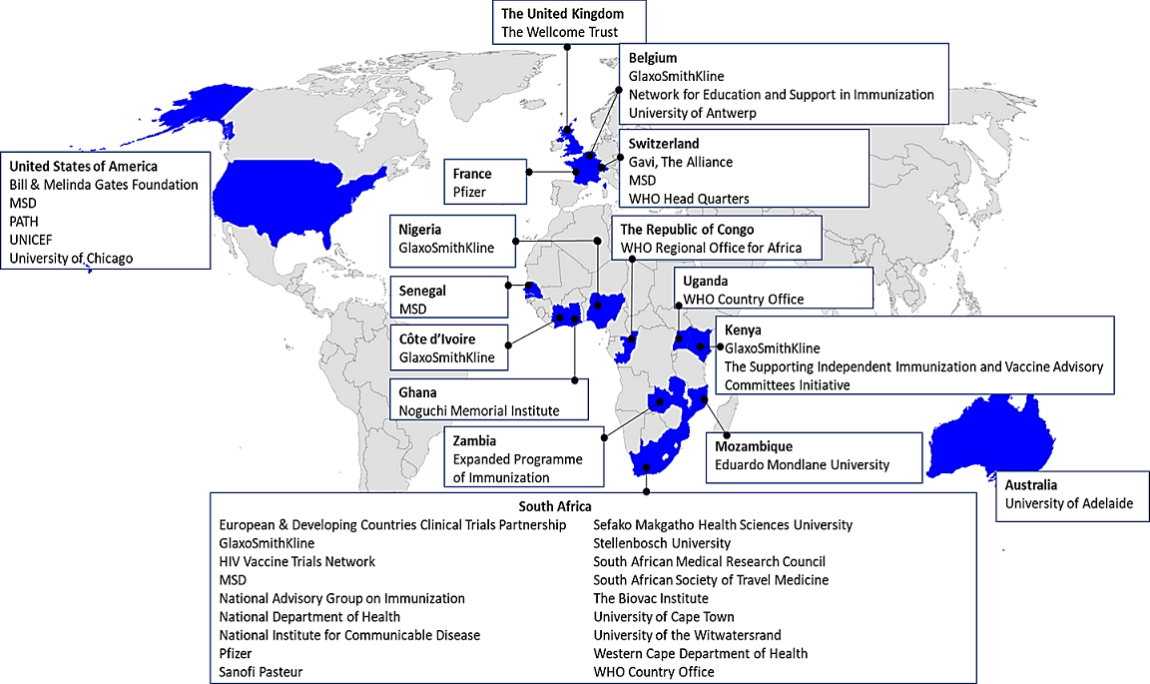
affiliations of faculty members of the Annual African Vaccinology Course between 2011 and 2019

**Course faculty members:** about 116 faculty members have given talks in the AAVC between 2011 and 2019. On average, 33 faculty members regularly give talks at the AAVC each year. The AAVC faculty is well-represented by local, regional and international experts from relevant fields across the vaccinology cascade, including academia, global health agencies, non-governmental agencies and the pharmaceutical industry (Figure 2). The diversity of the faculty allows for a rich pool of expertise in fields such as epidemiology, health economics, health policy, health systems, immunology, infectious diseases, national immunization programmes, paediatrics, vaccine communication and advocacy, as well as the vaccine development and supply chain process.

## Discussion

**Course evaluation:** to ensure that the AAVC continues to be responsive to the training needs of vaccinologists in Africa, the organizing committee places strong emphasis on course evaluations. Participants are invited to complete an online evaluation of the pre-course prior to attending the AAVC. In addition to this, a post-course feedback survey is also conducted to ascertain participants´ impression of the organization, course content and management of the AAVC. Altogether, such feedback is critical to improving the content and design of future AAVCs. A summary of the course evaluation administered to participants of the 15^th^ AAVC held in 2019 is provided in Annex 1. In general, participants indicated that completing the online pre-course reading prepared them well for the face-to-face meeting. In addition, feedback from the evaluation indicated that the 15^th^ AAVC was well organized and met the expectations of the participants. Broadly, the suggestions for future AAVCs included allowing more time for questions during the face-to-face meeting, adding podcast or listening materials to the online pre-course materials and allowing participants to attend the AAVC for a second time. There is a need to expand the course evaluation to include the impact of the course on national immunization programmes within the continent.

## Conclusion

One of the major challenges faced by the AAVC lies in meeting the growing demand for vaccinology training with the limited funding available. We therefore advocate for sustainable funding to support vaccinology training programmes within Africa. We plan to build on the success of the online pre-course to develop an e-vaccinology course. Resource permitting, the e-vaccinology course will be tailored with language translation options for participants from Francophone and Lusophone African countries who have been previously underserved. We anticipate that this online version of the course will complement the face-to-face format of the AAVC and expand the reach of vaccinology training in Africa. Such a blended approach has become even more imperative given the global restrictions on international travel and public gatherings in response to the COVID-19 pandemic in 2020. Other future considerations are to offer expert courses such as Evidence-Informed Decision-Making (EIDM) for vaccines and immunization, integration of health economics and mathematical modelling in EIDM, Covid-19 vaccine rollout, and vaccine safety monitoring. In addition, the AAVC may be adapted to meet specific training needs of NITAGs. As we move towards the Immunization Agenda 2030 [10], the AAVC aims to contribute to regional ownership of immunization programmes, by expanding the pool of trained vaccinologists and strengthening the scientific and technical capacity of NITAGs in Africa.

## Annex

**Annex 1**: summary of the 15^th^ AAVC evaluation questions
